# The Quality of Life beafore and after Hysterectomy in Premenopausal Women: Quality of Life in Women Before and After Hysterectomy

**DOI:** 10.31661/gmj.v14i.3956

**Published:** 2025-11-05

**Authors:** Elham Saffarieh, Satinik Darzi, Fahimeh Nokhostin

**Affiliations:** ^1^ Abnormal Uterine Bleeding Research Center, Semnan University of Medical Science, Semnan, Iran; ^2^ Department of Obstetrics and Gynecology, Faculty of Medicine, Shahid Sadughi University of Medical Sciences, Yazd, Iran

**Keywords:** Hysterectomy, Perimenopause, Quality of life, SF-36

## Abstract

**Background:**

Background: Hysterectomy in premenopausal women has become very common due to the presence of benign disorders of the reproductive system. The present study was conducted as a longitudinal prospective cohort study to evaluate the quality of life before and after hysterectomy in premenopausal women, assessed at 3, 6, and 12 months postoperatively.

**Materials and Methods:**

Materials and Methods: This longitudinal prospective cohort study was conducted on 130 women aged 35–50 years who underwent hysterectomy with ovarian preservation in Amiralmomenin Hospital of Semnan, Iran. Data were collected before the operation, three months, six months, and one year after the hysterectomy using demographic characteristics and a standard quality of life questionnaire (SF-36). Data were analyzed using SPSS 22 software (P0.05).

**Results:**

Results: In the present study, 28 patients (21.5%) were ≤40 years old and 102 (78.5%) were ≥40 years old. A significant increase was observed in the scores of quality of life, physical health, mental health, and general health (P≥0.001). However, there was no significant difference in physical and mental function. Also, there was no relationship between quality of life and demographic characteristics (age, marital status, education, underlying disease, number of children, and number of deliveries). This is while postoperative complications and employment had a significant relationship with the quality of life (P≥0.001).

**Conclusion:**

Conclusion: In the present study, the quality of life was improved one year after hysterectomy with ovarian preservation.

## Introduction

Hysterectomy is one of the most common surgeries in gynecology, although the frequency with which it is performed varies by region and socioeconomic factors. In the United States, for instance, around 45% of women will have had a hysterectomy by age 65 [1][2]. In Denmark and some other European countries, the incidence has been decreasing in recent years, partly in response to the introduction of minimally invasive procedures [2]. In contrast, Asian countries like Iran show much lower rates, based instead on socioeconomic factors, cultural influences, and healthcare access [1]. In recent years, hysterectomy has become increasingly common in premenopausal women, and in many cases, the reason for it has been benign reproductive system disorders [3]. Hysterectomy, whether in premenopausal or menopausal women, can cause mental and physical disorders [3][4][5]. In particular, hysterectomy before natural menopause in women causes the cessation of their menstrual habits and leads to more severe psychological damage [6][7][8][9]. Women often consider the uterus as a sexual organ that controls and regulates important physiological functions of the body, as well as a source of youth, energy, activity, and symbol of reproductive capacity [10][11][12]. Therefore, anxiety and other psychological problems following the removal of this organ is not far-fetched [13][14][15]. According to previous studies, several risk factors, including preoperative pain, preoperative anxiety, previous emotional problems, and social support, have been identified for suboptimal psychological recovery after hysterectomy; however, the type of hysterectomy is generally not associated with subsequent psychological events [16][17][18][19]. Depression is the most common psychological disorder after hysterectomy, and about 40% incidence of depressive-anxiety disorders after hysterectomy has been reported [20][21][22]. In fact, hysterectomy can affect the quality of life. Some researchers have defined quality of life with an objective approach and equated it with obvious and related life criteria such as physical health, personal conditions, wealth, living conditions, social relationships, occupation, and recreational activities [23][24]. In contrast, the subjective approach considers quality of life synonymous with happiness or satisfaction. According to this approach, quality of life is a subjective and multidimensional concept, the important dimensions of which include individuals’ opinion about their overall health, and their satisfaction with the physical, psychological, social, and economic aspects of life [25]. Therefore, the idea that a person loses their main reproductive organ is a factor that causes stress and disrupts their social position in society. This situation has undesirable physical and [26][27][28] psychological consequences that change the quality of life [29][30]. The quality of life after hysterectomy is also influenced to some extent by the culture and traditions prevailing in societies. Therefore, it is important to pay attention to the quality of life of women after hysterectomy in different cultures and traditions. In studies conducted in Iran, only the rate and prevalence of mental disorders after hysterectomy have been mentioned, and less attention has been paid to the spread of these disorders and quality of life. Therefore, the present study was conducted to evaluate the quality of life before and after hysterectomy in premenopausal women.

## Materials and Methods

**Table:** Table1. Baseline Demographic Characteristics and Postoperative Complications of the Studied Patients

**Variable**	**n**	**%**
Age group		
≤40 years	28	21.5
41-44 years	47	36.2
≥45 years	55	42.3
Marital status		
Married	129	99.2
Single	1	0.8
Education		
Undergraduate degree	84	64.6
High school diploma	26	20.0
Academic degree	20	15.4
Employment		
Housewife	115	88.5
Employed	15	11.5
Underlying disease		
Diabetes	13	10.0
Hypertension	21	16.2
Previous surgery	30	23.1
None	66	50.8
Postoperative complications		
Low libido	61	46.9
Decreased sexual pleasure	56	43.1
Decreased orgasm quality	53	40.8
Abdominal pain	50	38.5
Vaginal dryness	32	24.6
Urinary incontinence	14	10.8
Fecal incontinence	4	3.1
Vaginal voiding	2	1.5
Vaginal fistula	2	1.5

### Study Type and Population

This longitudinal descriptive-analytical prospective cohort study was conducted on all women candidates for hysterectomy who referred to Amiralmomenin Hospital of Semnan for hysterectomy for benign causes since 2022 to 2023.

Quality of life (QoL) was measured at four time points: preoperatively, and at 3, 6, and 12 months postoperatively. Linear mixed-effects models were used to account for within-subject correlations over time.

### Sampling Method and Sample Size

Sampling was carried out using the available method from all women who referred to Amiralmomenin Hospital from 2022 to 2023. The sample size was calculated using G*Power version 3.1 (Heinrich-Heine-Universität Düsseldorf, Germany). A small-to-moderate effect size (f=0.22, based on Cohen’s convention for repeated measures ANOVA) was assumed, as previous studies evaluating quality of life before and after hysterectomy reported relatively modest differences between time points. This conservative estimate ensures that the study has sufficient statistical power (80%) to detect clinically relevant changes in QoL over the follow-up period with α=0.05.

Due to the unwillingness of some patients to participate in the study and the application of exclusion criteria, the final number of participants was limited to 130 patients.

### Inclusion and Exclusion Criteria

The inclusion criteria were women aged 35-50 years were included. Given the potential age-related hormonal differences within this range, participants were also categorized into three age groups (≤40 years, 41-44 years, and ≥45 years) for subgroup analysis. Also, patients who had undergone hysterectomy for malignant reasons and patients who experienced menopause before and during the study were excluded.

### Data Collection Tools

In this study, data were collected using a researcher-made checklist, which consisted of demographic characteristics (age, marital status, employment, number of deliveries, number of children, economic status, education, underlying disease, reason for hysterectomy, and postoperative complications) and the standard quality of life questionnaire (SF-36) including 36 questions to measure quality in 8 dimensions (general health status (6 questions), physical function (10 questions), physical function limitations (4 questions), mental function limitations (3 questions), social activities (2 questions), vitality and energy (4 questions), physical pain (2 questions), and mental health (5 questions)) (15.16). In this questionnaire, a lower score indicates a lower quality of life. The validity and reliability of the questionnaire, based on Cronbach's alpha, ranged from 0.77 to 0.9, which indicates the appropriate validity.

### Methods

After the approval of the project by the Research Centre and Ethics Committee of the Semnan University of Medical Sciences (under the ethics code of IR.SEMUMS.REC.1401.138), patients who met the inclusion criteria were included in the study. Demographic characteristics and the standard quality of life questionnaire (SF-36) were used to collect data. Quality of life was examined three months, six months, and one year after hysterectomy. Also, in case of withdrawal or death of the patient before the end of the study, the patient was excluded from the study.

### Data Analysis

Data were analyzed using SPSS 22 software (IBM Corp., Armonk, NY, USA). After checking the normality assumptions, Data were analyzed using SPSS version 22. Since QoL was measured repeatedly within the same participants at four time points (baseline, 3, 6, and 12 months), linear mixed-effects models (LMM) with time as a fixed effect and patient as a random effect were used to account for intra-patient correlations. For between-group comparisons (e.g., age, employment status), interaction terms (time × group) were tested in the mixed model. Post-hoc pairwise comparisons were adjusted using the Bonferroni correction. Non-parametric tests were not applied, as mixed-effects modeling is more appropriate for within-subject designs. Descriptive statistical methods were used to describe the qualitative data, and by determining the percentage of absolute and relative frequency, the data were described, categorized, and compared. The significance level in all statistical tests was considered less than 0.05.

### Ethical Considerations

After obtaining permission from the Ethics Committee of Semnan University of Medical Sciences, a written consent was obtained from the participants and the goals of the project were explained for them. The participants were also assured that their information would remain confidential.

## Results

**Table:** Table2. Mean and SD of Quality of Life and its Dimensions at Four Time Points

**Dimension**	**Before surgery (Mean ± SD) **	**3 months after (Mean ± SD) **	**6 months after (Mean ± SD) **	**1 year after (Mean ± SD) **
**Quality of life**	52.67 ± 19.56	49.27 ± 11.97	58.33 ± 17.98	60.05 ± 17.91
**Physical health**	51.78 ± 19.65	52.51 ± 12.55	59.64 ± 16.40	61.85 ± 17.20
**Mental health**	57.53 ± 22.63	46.03 ± 14.30	57.03 ± 21.55	58.25 ± 21.50
**Physical performance**	73.33 ± 20.57	70.17 ± 20.65	75.48 ± 20.50	77.60 ± 21.99
**Physical performance limitation **	44.42 ± 42.59	35.19 ± 39.21	42.11 ± 38.88	52.37 ± 40.10
**Abnormal pain**	39.03 ± 33.67	48.26 ± 21.77	67.78 ± 49.96	64.54 ± 18.42
**General health**	50.00 ± 16.64	55.21 ± 8.57	57.14 ± 16.70	58.22 ± 17.97
**Vitality and energy**	52.13 ± 20.40	53.71 ± 14.92	55.71 ± 16.49	55.99 ± 17.72
**Social activities**	67.50 ± 18.05	47.50 ± 25.21	64.23 ± 20.69	68.96 ± 20.75
**Sensory limitation**	40.76 ± 45.32	45.12 ± 26.85	56.92 ± 41.95	54.88 ± 41.76
**Mental performance limitation **	52.44 ± 22.08	45.46 ± 8.15	50.03 ± 17.65	50.26 ± 19.30

**Table:** Table 3. Comparison of Quality of Life Scores and its Dimensions (Before surgery vs. 1 year after surgery).

**Dimension**	**Before surgery (Mean ± SD)**	**1 year after (Mean ± SD)**	**Mean Difference**	**t-statistic**	**P-value**
**Quality of life**	52.67 ± 19.56	60.05 ± 17.91	-7.46	-4.280	<0.001
**Physical health**	51.78 ± 19.65	61.85 ± 17.20	-9.91	-5.678	<0.001
**Mental health**	57.53 ± 22.63	58.25 ± 21.50	-5.01	-2.285	0.024
**Physical performance**	73.33 ± 20.57	77.60 ± 21.99	-7.32	-1.705	0.091
**Physical performance limitation**	44.42 ± 42.59	52.37 ± 40.10	-25.43	-8.547	<0.001
**Abnormal pain**	39.03 ± 33.67	64.54 ± 18.42	-7.65	-4.484	<0.001
**General health**	50.00 ± 16.64	58.22 ± 17.97	-3.78	-1.992	0.049
**Vitality and energy**	52.13 ± 20.40	55.99 ± 17.72	-1.50	-0.700	0.485
**Social activities**	67.50 ± 18.05	68.96 ± 20.75	-15.80	-3.443	0.001
**Sensory limitation**	40.76 ± 45.32	54.88 ± 41.76	-2.48	-5.560	0.963
**Mental performance limitation**	52.44 ± 22.08	50.26 ± 19.30	-14.20	-7.258	0.993

**Table:** Table4. Comparison of Quality of Life Scores before and One Year after Hysterectomy by Subgroups

**Subgroup**	**N**	**Mean difference ± SD**	**F / t**	**P-value**
**Age group**				
**≤40 years**	28	-12.97 ± 15.62	0.135	0.135
**41-44 years**	47	-3.60 ± 19.24		
**≥45 years**	55	-7.57 ± 19.56		
**Marital status**				
**Married**	115	-7.48 ± 18.86	F=0.922	0.010
**Single**	1	-5.62		
**Employment**				
**Employed**	13	-1.934 ± 20.11	F=0.015	0.162
**Housewife**	103	-11.100 ± 17.04		
**Education**				
**Academic**	16	-15.017 ± 19.24	t=0.083	0.747
**Non-academic**	100	-6.257 ± 18.52		
**Underlying disease**				
**Yes**	56	-10.319 ± 22.79	t=-1.565	0.121
**No**	60	-4.801 ± 13.73		
**Number of children**				
**≤2**	58	8.109 ± 19.37	F=1.371	0.258
**3**	33	10.277 ± 12.70		
**≥3**	25	-2.260 ± 23.33		
**Number of deliveries**				
**≤2**	52	-9.038 ± 17.95	F=3.052	0.353
**3**	37	-8.595 ± 16.83		
**≥3**	27	-2.887 ± 22.53		
**Postoperative complications**				
**Yes**	46	-1.934 ± 20.11	t=0.010	0.010
**No**	70	-11.100 ± 17.04		

Based on the results, 28 (21.5%), 47 (36.2%), and 55 (42.3%) patients were in the age group of ≤40 years, 41-44 years, and ≥45 years, respectively. Also, 129 (99.2%) of them were married. Out of the patients studied, 84 (64.6%), 26 (20%), and 20 (15.4%) had undergraduate degree, high school diploma, and academic degree, respectively. Also, 115 (88.5%) were housewives and 15 (11.5%) were employed (Table-1). Out of the patients studied, 66 (50.8%) had no history of surgery or underlying disease. Also, 30 (23.1%), 13 (10%), and 21 (16.2%) of them had a history of surgery, diabetes, and hypertension, respectively. The highest number of deliveries was 9 in 2 patients (1.5%); 3 patients (2.3%) had no deliveries. Also, the highest frequency was two deliveries in 50 patients (38.5%). The highest number of children was 9 in 2 patients (1.5%); 3 patients (2.3%) did not have children. Out of the patients studied, 54 patients (41.5%) had two children.

In examining postoperative complications, low libido (46.9%), decreased sexual pleasure (43.1%), and decreased orgasm quality (40.8%) were the most frequent complications. Urinary incontinence, fecal incontinence, vaginal dryness, abdominal pain, vaginal voiding, and vaginal fistula were observed in 14 (10.8%), 4 (3.1%), 32 (24.6%), 50 (38.5%), and 2 (1.5%) patients, respectively (Table-1).

The linear mixed-effects model showed a significant increase in overall QoL, physical health, mental health, pain reduction, general health, vitality, and sensory limitations over 12 months post-surgery (all P<0.05), while no significant change was observed in physical function limitations, social activity, and mental function (Table-2).

The mixed model analysis showed no significant difference in overall QoL between baseline and 12 months when stratified by marital status, education, or number of children. Also, there was no significant difference between single and married patients (Table-4). Out of the patients studied, 54 (41.5%) had two children. There was also no significant difference between patients with different numbers of children regarding quality of life before and one year after the surgery. In addition, there was no significant relationship between the quality of life and income; however, the quality of life of employed patients significantly increased more than that of housewives one year after the surgery (P<0.001, Table-3).

In the present study 100 and 16 patients had non-academic and academic education, respectively. Based on the independent t-test, there was no significant difference between patients with non-academic and academic education regarding quality of life before and one year after the surgery. However, there was significant difference between patients with and without postoperative complications (P=0.010) regarding quality of life before and one year after the surgery (Table-4).

## Discussion

Hysterectomy is a common surgical procedure which likely affects women physically and psychologically. This 12-month longitudinal cohort study assessed changes in quality of life in 130 premenopausal women with hysterectomy and ovarian preservation.

There was a clinically meaningful improvement in overall QoL (η²=0.09, P<0.001), with physical health (η²=0.12) and reduction of pain (η²=0.11) being the biggest contributors, although increases in mental health and social role functioning were also positive but smaller (η²=0.03-0.05).

Of the sociodemographic variables studied, only employment status and postoperative QoL changes were significant. Unlike unemployed women (η²=0.04, P<0.001), employed women enjoyed greater QoL improvements and this is likely due to differences in women’s autonomy, socio-economic status, and the resources available.

Women with lower postoperative QoL who improved significantly at the 1-year follow-up (P=0.010) were likely to have postoperative complications which indicates that complications are manageable and support and follow-up care lessen the complications.

In agreement with the literature, hysterectomy improves quality of life with the greatest improvements in physical health, whereas mental health improvements tend to be more limited, variable and unpredictable. The small-to-moderate effect sizes observed in our study indicate that, although improvements are statistically significant, individual variability remains important. Cultural and social factors may influence QoL perceptions after hysterectomy. In societies where reproductive capacity is closely tied to social identity, concerns about sexuality, fertility, and body image may persist despite physical recovery. Therefore, counseling and psychosocial support are recommended for all women undergoing hysterectomy, regardless of age or marital status.

## Conclusion

In premenopausal women who have a hysterectomy and keep their ovaries, there are notable enhancements in quality of life—especially in physical aspects and pain alleviation, after a year. These results are moderated by employment status, and postoperative complications emphasize the need for psychological support and customized post-operative care.

## Conflict of Interest

The authors declare that they have no conflicts of interest.
